# Nosocomial bloodstream infection and the emerging carbapenem-resistant pathogen *Ralstonia insidiosa*

**DOI:** 10.1186/s12879-019-3985-4

**Published:** 2019-04-23

**Authors:** Qingqing Fang, Yu Feng, Ping Feng, Xiaohui Wang, Zhiyong Zong

**Affiliations:** 1 Center of Infectious Diseases, West China Hospital, Sichuan University, Chengdu, China; 2 Division of Infectious Diseases, State Key Laboratory of Biotherapy, Chengdu, China; 3 Department of Infection Control, West China Hospital, Sichuan University, Chengdu, China

**Keywords:** *Ralstonia insidiosa*, Carbapenem-resistant, Bloodstream infections, Nosocomial infection, Whole genome sequencing

## Abstract

**Background:**

*Ralstonia picketti*, *Ralstonia mannitolilytica,* and *Ralstonia insidiosa* have recently been regarded as emerging pathogens of infectious diseases, in particular as the pathogens responsible for nosocomial infection in immunocompromised patients. *R. insidiosa* differs from *R. picketti* and *R. mannitolilytica*, and its related infections are rarely reported.

**Methods:**

Clinical data from two nosocomial bloodstream infection cases were extracted and analyzed. The causable isolates were identified by the VITEK 2 Compact system, matrix assisted laser desorption ionization-time of flight mass spectrometry (MALDI-TOF MS), and molecular identification methods using PCR with universal and species-specific primers. Antimicrobial susceptibility testing was performed using the broth microdilution method. Both of the isolates were subjected to whole genome sequencing using a HiSeq X10 Sequencer. Antimicrobial resistance genes, virulence factors, and plasmid replicons were identified from assembled genomes. A real-time RT-PCR experiment and a cloning experiment were conducted to explore the related class D β-lactamase-encoding genes.

**Results:**

Both patients recovered under therapy with antibiotics. Isolates were initially misidentified as *R. mannitolilytica* by the VITEK 2 Compact system rather than *R. insidiosa,* as identified by both MALDI-TOF MS and 16S rRNA gene sequencing. Both isolates were resistant to aminoglycosides, β-lactams, and polymyxin B. One isolate harboring *bla*_OXA-570_ was resistant to carbapenems. The whole genome sequencing data confirmed species identification based on average nucleotide identity (ANI) and revealed two variants of class D β-lactamase-encoding gene *bla*_OXA_ (*bla*_OXA-573_ and *bla*_OXA-574_). The real-time RT-PCR experiment showed no difference in gene expression between *bla*_OXA-570_ and *bla*_OXA-573_ in our strains. The cloning experiment showed that variant OXA-573 had no carbapenem hydrolase activity.

**Conclusions:**

We described two cases of nosocomial bloodstream infection caused by *R. insidiosa* strains. MALDI-TOF MS was cost-effective for rapid species identification. Clinicians should be aware that *R. insidiosa* can be resistant to commonly used antibiotics, even carbapenems.

## Background

The six effectively published species defining type strains of the genus *Ralstonia* (*Ralstonia insidiosa*, *Ralstonia mannitolilytica*, *Ralstonia picketti*, *Ralstonia pseudosolanacearum*, *Ralstonia solanacearum,* and *Ralstonia syzygii*) are aerobic gram-negative, non-fermentative, and rod-shaped bacteria. The bacteria are usually isolated from plants and soils. *R. picketti*, *R. mannitolilytica* and *R. insidiosa* have been recently regarded as pathogens of infectious diseases, especially as causable agents of nosocomial infection related to immunocompromised patients [1]. Contaminated solutions or water are believed to be the sources of *Ralstonia* nosocomial infections [1, 2]. The previous studies showed that most cases of infection were caused by *R. picketti*, with a few caused by *R. mannitolilytica*. The pathogens may cause bloodstream infection, pneumonia, peritonitis, meningitis, endocarditis, spinal osteitis, osteomyelitis, septic arthritis, and prostatitis. *R. insidiosa* was proposed as a new species in 2003 [3]. Bloodstream infection-related cases are extremely rare; none have been reported from Asia. In addition, further information concerning *R. insidiosa*, such as antimicrobial susceptibility, genomic profile and epidemiology, is also limited. Such information is clinically relevant, as *R. insidiosa* may have been underdiagnosed for the past several decades due to its low incidence. The bacterium could also easily be overlooked by researchers. Hence, in this study we described two cases of nosocomial bloodstream infection caused by *R. insidiosa* strains, including a carbapenems-resistant type.

## Methods

### Data collection

Two cases occurred among inpatients in an orthopedics ward at a 5000-bed tertiary hospital in western China. The clinical data were retrieved from medical records and included demographics, symptoms, physical examination findings, laboratory results, comorbidities, treatments, and outcomes. Three physicians independently reviewed the data and defined both cases as nosocomial bloodstream infections.

### Isolation and identification

During hospitalization, when fever was noticed, two sets of peripheral blood cultures were collected in both aerobic and anaerobic bottles (BioMérieux, Marcy-l’Étoile, France). Secondary samples cultured in brucella agar plates supplemented with defibrinated sheep blood (5% *v*/v) were incubated aerobically at 35 °C for up to 48 h. Isolates were initially identified with both the VITEK 2 Compact system (BioMérieux, Marcy-l’Étoile, France) and matrix-assisted laser desorption ionization-time of flight mass spectrometry (MALDI-TOF MS) (IVD MALDI Biotyper system, Microflex LT/SH, Bruker, Billerica, MA). For automatic biochemical identification, VITEK® 2 GN card and VITEK® 2 systems software version 7.01 were used. For mass spectrometry identification, Microflex LT was used to collect spectra, and the software FlexControl 3.4 (Bruker Daltonik) was used to analyze data.

Further species identification was established using partial 16S rRNA gene sequencing with universal primers 27F/1492R and species-specific primers Rp-F1/R38R1 [4]. Nucleotides of amplicons were compared against those of *R. insidiosa* strain ATCC 49129 and FC1138 using the BLASTn algorithm (http://blast.ncbi.nlm.nih.gov/Blast.cgi).

### Antimicrobial susceptibility testing

The minimum inhibitory concentrations (MICs) of 18 antimicrobial agents, which were automatically performed by VITEK 2 Compact system, were manually confirmed using the broth microdilution method (Table 1). *Pseudomonas aeruginosa* ATCC 27853 and *Escherichia coli* ATCC 25922 were tested alongside as quality control strains. Interpretation of MIC results was made following the recommendations of breakpoints for *Pseudomonas* spp., *Burkholderia cepacia,* and *Acinetobacter* spp. from the Clinical and Laboratory Standards Institute (CLSI) [5].

**Table 1:** Resistance patterns of two *R. insidiosa* isolates

Antibiotics	WCHRI065437(μg/mL)	WCHRI065162(μg/mL)
Amikacin	64(R)	128(R)
Amoxicillin/Clavulanate	32(R)	32(R)
Ampicillin	32(R)	32(R)
Aztreonam	256(R)	512(R)
Cefepime	2(S)	16(I)
Ceftazidime	8(S)	16(I)
Ceftriaxone	0.5(S)	0.5(S)
Ciprofloxacin	< 0.125(S)	< 0.125(S)
Gentamicin	32(R)	16(R)
Imipenem	1(S)	8(R)
Levofloxacin	0.125(S)	0.25(S)
Meropenem	1(S)	8(R)
Nitrofurantoin	512(R)	512(R)
Piperacillin/Tazobactam	< 4/4(S)	< 4/4(S)
Polymyxin B	> 512(R)	> 512(R)
Tigecycline	0.5(S)	1(S)
Tobramycin	32(R)	32(R)
Trimethoprim/Sulfamethoxazole	< 0.25/4.75(S)	< 0.25/4.75(S)

R, resistant; S, susceptible; I, intermediate

### Whole genome sequencing, species confirmation and phylogenetics

Isolates were cultured overnight in 50 mL BHI broth. Genomic DNA was extracted using a DNeasy Blood & Tissue Kit (QIAGEN, Hilden, Germany). Quantification and quality assays were performed using a Qubit 2.0 fluorometer and a NanoDrop 2000/2000c spectrophotometer. The extracted DNA was sheared into 350 bp ultrasonically prior to a 150 bp paired-end library construction and then sequenced using a HiSeq X10 Sequencer (Illumina, San Diego, CA, USA). The raw sequencing reads of isolates WCHRI065162 and WCHRI065437 were trimmed using Trimmomatic v0.38 [6] prior to being assembled into draft genomes using SPAdes v3.12.0 [7] under the careful mode. The sequences were deposited into GenBank with assigned accession numbers PKPC00000000 and PKPB00000000, respectively. Antimicrobial resistance genes, virulence factors, and plasmid replicons were identified using whole genome data by the program ABRicate v0.8 (https://github.com/tseemann/abricate). Genome-based species identification was performed by comparing the average nucleotide identity (ANI) against each existing species of genus *Ralstonia* using the web program Jspecies (http://imedea.uib-csic.es/jspecies/). The relatedness between strains was described by the number of single nucleotide polymorphisms (SNPs) in the core genome identified using Roary v3.11.2 [8] with default settings. The core SNP-based phylogenetic tree was inferred using RAxML v 8.2.12 [9] under the GTRGAMMA model with 1000 bootstrap resamples. Comparative analysis was performed among genomes of our isolates and published *R. insidiosa* strains ATCC 49129 and FC1138. Using ATCC 49129 as the reference, a circular map was generated by the BLAST Ring Image Generator (BRIG) [10].

### Real-time RT-PCR and cloning experiments to explore the related class D β-lactamase-encoding genes

A real-time RT-PCR experiment was conducted to compare gene expression of *bla*_OXA-60-like_ at the mRNA level in our two strains. Total RNA was obtained using an RNAprep pure Cell/ Bacteria Kit (TIANGEN BIOTECH, Beijing, China). The concentrations and quality of RNA were determined by measuring the absorbance at 260 nm. A PrimeScript™ RT reagent Kit with gDNA Eraser (Takara Bio, Beijing, China) was used for reverse transcriptase PCR (RT-PCR). Real-time RT-PCR was performed using primers described in Table 2 and the house-keeping gene *rpoB* as the internal control. Amplification was carried out in a 25 μL-final-volume containing 12.5 μL TB Green Premix Ex taqII mix, 1 μL primers (5 nM each), 8.5 μL H_2_O, and 2 μL cDNA. The running protocol was denaturation at 95 °C for 90 s, followed by 40 cycles of denaturation at 95 °C for 10 s, annealing at 52 °C for 10 s, and elongation at 72 °C for 30s. The 2^-∆∆*C*^_T_ method [11] was used to quantify the exact ratio of the genes.

**Table 2:** Primers used for amplification and sequencing

Primer	Sequence (5′ → 3′)
OXA569-up-EcoRI	AACGAATTCATGATGAAACTCCGCCACGC
OXA569-down-SacI	AACGAGCTCAAATCAGTGACTCGCAAGGGCCA
OXA570-up-EcoRI	AACGAATTCATGTTTGCTCGCTGGTCAAA
OXA570-down-SacI	AACGAGCTCAAACTAGGGTGTTGGCCACA
OXA573-up-EcoRI	AACGAATTCATGTTCCCTCGCTGGTCAAA
OXA573-down-SacI	AAAGAGCTCAAACTAGGGTGTTGGCCAC
OXA574-up-EcoRI	AACGAATTCATGATGAAACTCCGCCACGC
OXA574-down-XhoI	AACCTGCAGAAATCAGTGGCTCGCAAGGGC
M13 R	GTAAAACGACGGCCAGT
M13 F	CAGGAAACAGCTATGACC
OXA570-RT-F	TCAAGCGCACGCCGAGTTGA
OXA570-RT-R	GGCACCCGTATCGAAGGC
OXA573-RT-F	ATGACCTCAAGCGCGTGTTC
OXA573-RT-R	GCACCCGTATCGAAGGC
RpoB-R.in-F	GTCCATCAGGTTCCCTTCC
RpoB-R.in-R	GGACAGGTGATACGACACGA

^a^Restriction sites located in primers are underlined

A cloning experiment was conducted to assess functionality of related class D β-lactamase-encoding genes in our two strains. The host cell was *Escherichia coli* DH10B (CUNMAI Biotechnologies, Shanghai, China). The cloning and expression vector was a chloramphenicol-resistant plasmid pBC-SK (NovoPro Biotechnology, Shanghai, China). According to a standard directional cloning molecular technique [12], the *bla*_OXA-60-like_ genes (*bla*_OXA-570_, *bla*_OXA-573_) and the *bla*_OXA-22-like_ genes (*bla*_OXA-569_, *bla*_OXA-574_) found in our strains were amplified using self-designed primers (Table 2), followed by a restriction digestion using selected endonucleases. Recombinant plasmids were transformed into competent *E. coli* DH10B cells after ligation by the heat-shock method*.* The transformed recipient cells were challenged on the chloramphenicol (40 μg/mL) Luria-Bertani (LB) plates for 16 h at 37 °C. Selected colonies were amplified using the universal primer M13 and sequenced to ensure that no mutation occurred. All *E. coli* DH10B strains harboring recombinant plasmids expressing OXA-570 (pBC1), OXA-573 (pBC2), OXA-569 (pBC3) and OXA-574 (pBC4) were tested for antimicrobial susceptibility to amoxicillin, ampicillin, cefepime, cefoxitin, ceftazidime, imipenem and meropenem using the broth microdilution method mentioned above. An *E. coli* DH10B strain without the transformed plasmid was included as a reference.

## Results

### Clinical information of case 1

A 43-year-old male was diagnosed with open multiple fractures and neurovascular injury in the left tibia, fibula, and radius. The patient immediately received debridement on the left leg and left hand. The patient underwent subsequent neurovascular exploration, reconstructive surgical procedures, and left tibia fracture decompression and fixation on the first admission day. On the seventh day, fever with a peak of 39.0 °C and a hematoma in the left elbow and forearm with severe exudation emerged. The leukocyte count increased to 11.18 × 10^9^/L with an elevated neutrophil proportion (88.8%). Mild abnormal hepatic dysfunction was noticed; alanine aminotransferase was 82 IU/L (reference range < 50 IU/L), and aspartate aminotransferase was 83 IU/L (reference range < 40 IU/L). Prior to incision and drainage, peripheral blood samples were sent to the clinical microbiology lab for culturing. Ceftazidime was given empirically and intravenously as 2000 mg every 12 h. The blood culture reported a positive result after 19 h of incubation, and this strain, designated as WCHRI065437, was then identified as *R. mannitolilytica* by the VITEK 2 Compact system (strain name WCHRI065437). The antimicrobial therapy was adjusted to ceftazidime 2000 mg every 8 h for 2 weeks. Finally, the patient completely recovered and was discharged.

### Clinical information of case 2

A 35-year-old male who had received a biopsy and chemotherapy in the past was admitted for surgery due to left femur osteosarcoma. The subsequent operations involved resection of a distal femoral tumor, assembled tumor knee replacement, neurovascular exploration and muscle reconstruction. A fever peaking at 38.7 °C without a focal infection was noticed on the fourth day. Blood tests revealed WBC 4.45 × 10^9^/L (neutrophil 87%), elevated C-reactive protein of 12.20 mg/L and IL-6 of 12.37 pg/mL. Alanine aminotransferase was mildly elevated to 97 IU/L (reference range < 50 IU/L), and aspartate aminotransferase was mildly elevated to 75 IU/L (reference range < 40 IU/L). Empirical intravenous antibiotic therapy with ciprofloxacin 400 mg per 12 h was immediately initiated. Peripheral blood culture revealed a bacterium designated as WCHRI05162 after 26 h of incubation that was also identified as *R. mannitolilytica* by the VITEK 2 Compact system. However, the leukocyte count kept climbing to 13.9 × 10^9^/L despite 3 days’ treatment. Since additional disc diffusion tests showed that the bacterium was susceptible to cefoperazone/sulbactam (38 mm), cefoperazone/sulbactam 1.5 g every 12 h was added. The patient became completely afebrile 2 days later and was transferred to a rehabilitation hospital.

### Isolates identification

Medium sized, round shaped, moist raised light brown colonies with neat edges were observed. Although the two isolates were misidentified as *R. mannitolilytica* by the VITEK 2 Compact system, they were accurately identified as *R. insidiosa* by both MALDI-TOF MS and 16S rRNA gene sequencing. Using either the universal primers 27F/1492R or the species-specific primers Rp-F1/R38R1, the nucleotide identity was 99%.

### MIC determination

Both strains were resistant to amikacin, amoxicillin/clavulanate, ampicillin, aztreonam, gentamicin, polymyxin B, tobramycin, and nitrofurantoin, but susceptible to ciprofloxacin, levofloxacin, ceftriaxone, piperacillin/tazobactam, tigecycline, and trimethoprim/sulfamethoxazole. Isolate WCHRI065162 from case 2 was resistant to imipenem and meropenem and was intermediate to cefepime and ceftazidime, whereas isolate WCHRI065437 was susceptible to all of them (Table 1).

### Whole genome sequencing, species confirmation, and phylogenetics

**Fig. 1:**
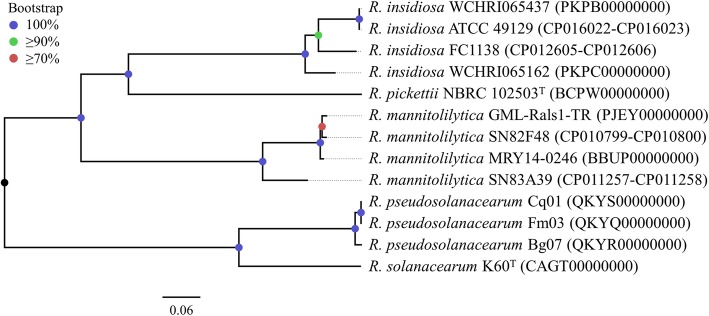
Phylogenetic tree based on core genome SNP. The tree was inferred using the maximum likelihood method under the GTRGAMMA model with a 1000-bootstrap test. Support values are colored based on confidence at internal nodes

**Fig. 2:**
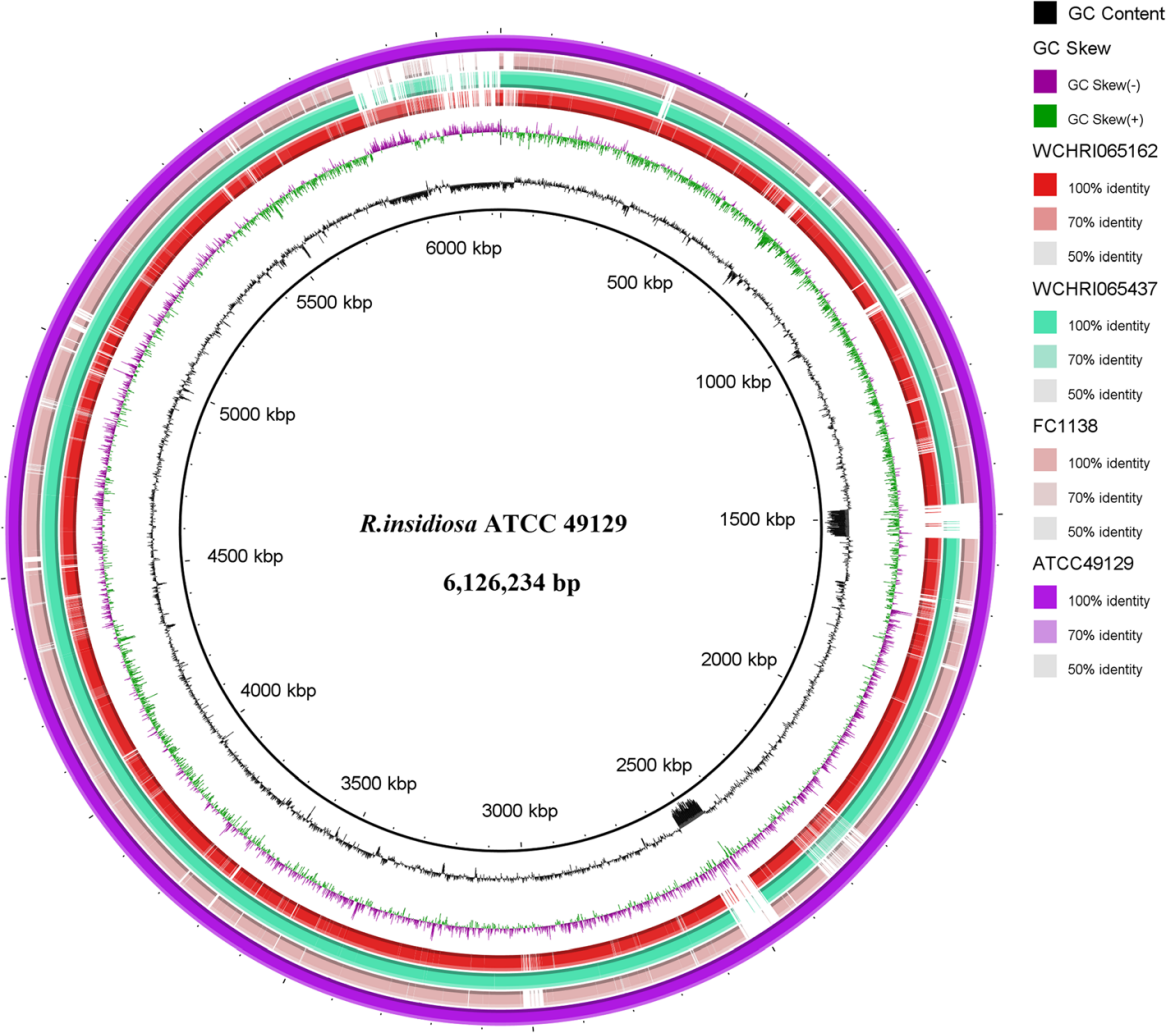
Comparative analysis among *R. insidiosa* genomes of our isolates and previously published strains ATCC 49129 and FC1138

The genome sizes revealed by the draft genomes of *R. insidiosa* strains WCHRI065162 and WCHRI065437 were 5,923,114 bp and 6,113,916 bp, respectively. Their G + C contents were 63.41 and 63.63%, respectively (Table 3). To confirm the results of rough species identification based on individual gene sequencing, ANI calculations were performed between two isolates and type strains of *R. picketti* and *R. solanacearum*. The results were interpreted according to the suggested threshold [13], showing that neither strain belonged to these two species. However, lacking genomic data of type strains of species *R. insidiosa*, *R. mannitolilytica*, *R. pseudosolanacearum* and *R. syzygii* in the public database, a phylogenetic analysis was performed on a total of 13 strains, including nine non-type strains of these four species, two publicly available type strains, and two from this study, to determine the closest related species of strains WCHRI065162 and WCHRI065437. Having determined that both isolates clustered with species *R. insidiosa* with high bootstrap values on the phylogenetic tree (Fig. 1), we observed that none of results from pairwise ANI calculations between the two isolates and all other genomes exceeded 95%, except for those calculated with *R. insidiosa* ATCC 49129 and FC1138 (Table 4). The results from the BLASTn algorithm of previous amplicon sequencing confirmed that WCHRI065162 and WCHRI065437 were strains of the species *R. insidiosa*. There were 74,392 bp SNPs between our two isolates, suggesting they were not the same clone. The circular map (Fig. 2) showed that several fragments were absent in our isolates compared to the chromosome sequences of *R. insidiosa* ATCC 49129 and FC1138.

**Table 3:** Genome information of two *R. insidiosa* isolates in this study

Isolate	*R. insidiosa* WCHRI065162	*R. insidiosa* WCHRI065437
total contig	53	47
total sequence (bp)	5,925,661	6,116,439
N50(bp)	329,971	910,997
G + C%	63.41	63.63
coding DNA sequence	5549	5717
Largest contig (bp)	602,553	1,542,049
CDS	5549	5717
tRNAs	54	55
rRNAs	3	3
tmRNA	1	1
class D β-lactamase gene	*bla*_OXA-569_、*bla*_OXA-570_	*bla*_OXA-573_、*bla*_OXA-574_
GenBank accession No.	NZ_PKPC00000000.1	NZ_PKPA00000000.1

**Table 4:** Calculated average nucleotide identity (ANI) between WCHRI065162, WCHRI065437 and other published strains of genus *Ralstonia*

Strains	*R. insidiosa* WCHRI065162 (%)	*R. insidiosa* WCHRI065437 (%)
*R. insidiosa* WCHRI065162	–	96.70
*R. insidiosa* WCHRI065437	96.70	–
*R. insidiosa* FC1138	96.65	97.09
*R. insidiosa* ATCC 49129	96.50	99.67
*R. pickettii* NBRC 102503^T^	85.98	85.93
*R. mannitolilytica* SN82F48	85.86	85.70
*R. mannitolilytica* SN83A39	85.70	85.66
*R. mannitolilytica* MRY14–0246	85.62	85.88
*R. mannitolilytica* GML-Rals1-TR	85.48	85.91
*R. solanacearum* K60^T^	84.33	84.19
*R. pseudosolanacearum* Fm03	83.66	83.62
*R. pseudosolanacearum* Bg07	83.65	83.64
*R. pseudosolanacearum* Cq01	83.64	83.61

We identified multiple putative virulence factors, including those involved in quorum sensing, biofilm formation, the production of bacteriocins, adhesin and invasins based on genome annotation. Both isolates contained seven quorum sensing and biofilm formation genes (*pelA*, *pelB*, *pelC*, *pelD*, *pelE*, *pelF*, and *pelG*) involved in glucose-rich biofilm formation; these were slightly different between the two isolates due to amino acid substitutions (3 in PelA, 11 in PelB, 2 in PelC, and 4 in PelD). Adhesion genes related to autotransporter adhesin, sigma-fimbriae tip adhesin, fimbrial adhesin and type V secretory pathway adhesin AidA were found in both isolates. Secretion systems related to adhesin, biofilm formation, and pilus assembly were also found. WCHRI065162 contained T2SS, T4SS, T5SS, T6SS and T7SS (the type II, IV, V, VI, VII secretion systems), while WCHRI065437 only contained T2SS, T4SS, T6SS and T7SS (the type II, IV, VI, VII secretion systems). Compared to *R. insidiosa* ATCC 49129 and FC1138, our isolates did not have the *hcp* gene. Both isolates harbored genes related to colicin V and the bacteriocin production cluster.

WCHRI065437 carried two novel variants of the gene encoding class D β-lactamase (*bla*_OXA-573_, *bla*_OXA-574_, accession numbers MG736320 and MG736321, respectively) which we deposited into the Bacterial Antimicrobial Resistance Reference Gene Database (https://www.ncbi.nlm.nih.gov/bioproject/PRJNA313047). WCHRI065162 carried two genes encoding class D β-lactamase (*bla*_OXA-569_ and *bla*_OXA-570_, accession numbers MG736316 and MG736317). OXA-569 and OXA-574 were highly similar to OXA-22 (amino acid identity 85 and 86%, respectively). The proteins OXA-570 and OXA-573 were also highly similar to OXA-60 (amino acid identity 84 and 86%, respectively). The promoter of *bla*_OXA-573_ was 46 bp shorter than that of *bla*_OXA-570_. All of these *bla*_OXA_ alleles were located on the chromosomes without transposable elements nearby, suggesting that they are intrinsic in *R. insidiosa*. None of the known plasmid replicons were found in either of the two isolates.

### Real-time RT-PCR and cloning experiments to explore the related class D β-lactamase-encoding genes

There was no significant difference between *bla*_OXA-570_ copy numbers in *R. insidiosa* WCHRI065162 and *bla*_OXA-573_ copy numbers in *R. insidiosa* WCHRI065437 (fold difference = 0.79), suggesting difference in gene expression at the mRNA level was not responsible for the carbapenem-resistant phenotype. However, the MICs of recombinant harboring plasmids pBC1 carrying *bla*_OXA-570_ against carbapenem and other β-lactams were significantly higher than those of reference strain *E. coli* DH10B (Table 5). There were no significant differences in antimicrobial susceptibility against cefepime, ceftazidime, imipenem or meropenem between *E. coli* DH10B and other recombinants harboring plasmids pBC2 (*bla*_OXA-570_), pBC3 (*bla*_OXA-569_), pBC4 (*bla*_OXA-574_) (Table 5). This suggested that in our strains only OXA-570 showed carbapenem hydrolase activity, which may be responsible for the carbapenem-resistance of isolate WCHRI065162.

**Table 5:** MICs to β-lactam antibiotics of *E. coli* DH10B and recombinant harboring plasmids pBC1, pBC2, pBC3 and pBC4

β-lactam	*E.coli*				
Antibiotics	DH10B	pBC1	pBC2	pBC3	pBC4
Meropenom	0.03	8	0.03	0.03	0.03
Imipenem	0.5	2	0.5	0.5	0.5
Cefepime	< 0.03	0.06	< 0.03	< 0.03	< 0.03
Cefoxitin	1	8	4	4	4
Ceftazidime	< 0.125	0.5	< 0.125	< 0.125	< 0.125
Ampicillin	8	256	8	16	16
Amoxicillin	4	128	4	16	16

pBC1: plasmid pBC-SK carrying *bla*_OXA-570_; pBC2: plasmid pBC-SK carrying *bla*_OXA-573_; pBC3: plasmid pBC-SK carrying *bla*_OXA-569_; pBC4: plasmid pBC-SK carrying *bla*_OXA-574_

## Discussion

Few cases of infections caused by *R. insidiosa* have been reported due to the limited awareness of the pathogen*. Ralstonia* spp. are emerging as global opportunistic pathogens affecting immunocompromised patients. Even without person-to-person transmission, the prevalence of *Ralstonia* infection is notably increasing. The bacteria reproduce in wet conditions and can survive in harsh environments (even in weak disinfectants) for long periods. The species exist widely in external aqueous environments including municipal water and medical water purification systems [2, 14]. As the bacteria can pass through 0.2-μm filters during the sterilization process, medical products may be contaminated during the manufacturing phase [15]. The species can create biofilms on the surfaces of medical supplies and produce toxins. Many infectious cases caused by *R. picketti* and *R. mannitolilytica* are due to the use of contaminated solutions, blood products, chlorhexidine, saline solution, and sterile water as well as the colonization of medical devices (tap water and water used for hemodialysis, bronchoscope flushing, and heparin for flushing) [16–18].

We described two rare nosocomial cases of bloodstream infections caused by *R. insidiosa*, although we could not track their source. Both of our patients were at a high risk for infection. One patient had a malignant tumor of the femur and underwent chemotherapy and surgery, while the other patient was bedridden for a long time because of severe multiple fractures and several surgical operations. High risk factors listed in the literature are cancer, blood vessel catheters, chronic renal failure, ischemic heart disease, newborn and other immunocompromised conditions [1]. The common symptom in these two cases was obvious fever, while the common abnormal routine test results were increased neutrophils and aminotransferase.

Based on our results, we recommend MALDI-TOF MS for rapid identification of *R. insidiosa*. Due to the inaccurate biochemical reactions, both of our *R. insidiosa* isolates were misidentified as *R. mannitolilytica* by the VITEK 2 Compact system, a widely accepted commercial automated biochemical identification instrument. The manual identification keys are that *R. insidiosa* metabolizes nitrate but not mannitol or arabinose, *R. picketti* metabolizes nitrate and arabinose but not mannitol, and *R. mannitolilytica* metabolizes only mannitol but not nitrate or arabinose [3]. *Ralstonia* spp. are very close to the *Burkholderia cepacia* complex and *Pseudomonas fluorescens*; the previous name of *R. picketti* was *Burkholderia pickettii*. Previously, we reported that the VITEK 2 system misidentified *Burkholderia pseudomallei* as *Burkholderia cepacia* [19]. This reminds us that clinicians should be cautious with automated identification of non-fermentative gram-negative bacilli, especially *Burkholderia* reported by the VITEK 2 system. For species identification, our study showed that using either the universal primers 27F/1492R or the species-specific primers Rp-F1/R38R1, 16S rRNA gene sequencing were practical. Additionally, species-specific primers Rp-F1/R38R1 were much more rapid for identifying *R. insidiosa* from *R. picketti* and *R. mannitolilytica*, because no amplicon band of *R. picketti* or *R. mannitolilytica* could be observed on the electrophoresis gel. However, considering turnaround time and PCR identification cost, MALDI-TOF MS would be a better choice. Our results supported the general consensus that MALDI-TOF MS could provide rapid and accurate results [20].

Both of our *R. insidiosa* isolates were multi-drug resistant, even the strain that was carbapenems-resistant. The antibiotic susceptibility data for *R. insidiosa* are very limited, not to mention that of clinical isolates. A study reporting antibiotic susceptibility profiles of 15 environmental *R. insidiosa* isolates showed that quinolones and the folate pathway inhibitors (sulfamethoxazole/trimethoprim) were most effective [21]. MICs results of our clinical isolates were consistent with those results and revealed that piperacillin/tazobactam and tigecycline were also susceptible alternatives. Considering accessibility, cost and side effects, quinolones may be a good choice for initial empirical therapy. However, since antimicrobial susceptibility (i.e., carbapenem susceptibility) discriminated between isolates, we still recommend that clinicians select antibiotics according to their MICs results.

Genome sequencing of our *R. insidiosa* WCHRI065162 and WCHRI065437 has improved current understanding of the antibiotic resistance and pathogenicity of this species. Due to a wide range of SNPs, our isolates were not the same clone, and neither of our isolates was close to those two fully assembled genomes of *R. insidiosa* in the NCBI database. Bacterial secretion systems present on the cell membranes are the cellular components used by pathogenic bacteria to secrete their virulence factors in order to invade the host cells. Although the classification is not very clear or complete, pathogenic gram-negative bacteria possess at least six secretory systems related to virulence factors [22]. The T2SS (Type II secretion system) contains a pseudopilus and secretes toxins and hydrolyzing enzymes. A main role of T4SS (type IV secretion system) is to facilitate the spread of drug-resistant genes located on plasmids through conjunction. T5SS (type V secretion system) is mainly involved in virulence factor secretion, intercellular adhesion and biofilm formation. The T3SS (type III secretion system), especially type IV pilus (T4P) biogenesis system, and T6SS (type VI secretion system) are now recognized as pathogenicity hallmarks in many gram-negative bacteria [23, 24]. These systems deliver bacterial proteins called effectors into neighboring bacteria or host cells, leading to cytotoxicity and cell death of targets. Both *R. insidiosa* ATCC 49129 and *R. insidiosa* FC1138 have T3SS and T6SS gene clusters, but our two isolates carried only T6SS gene clusters, implying that T6SS may be more crucial for the virulence of *R. insidiosa*. T7SS (type VII secretion system), rarely found in gram-negative bacteria, is a specialized secretion apparatus required for the virulence of mycobacteria [22]. However, all of *R. insidiosa* ATCC 49129, FC1138 and our isolates have T7SS. Further fundamental research is needed to reveal the role of T7SS in the virulence of *R. insidiosa*.

Quorum sensing, an efficient bacterial cell-to-cell communication process for population growth, involves bioluminescence, secretion of virulence factors, production of public products, and biofilm formation [25]. Both of our isolates contained biofilm formation genes (*Pel*) related to the generation of glucose-rich extracellular polysaccharide (PEL) and the construction of biofilm matrix. To some extent, these genes may be responsible for pathogenicity and multi-drug resistance phenotypes of *R. insidiosa*. Bacteriocin, one family of microbial defense systems, is now considered to be the most diverse and naturally abundant antimicrobial molecule [26]. Colicin V and bacteriocin production cluster subsystems were found in both of our isolates.

Both of our isolates were resistant to the β-lactam antibiotics, polymyxin B, and aminoglycosides. It is known that two chromosomally encoded class D β-lactamases, OXA-22 and OXA-60, are related to multidrug-resistance of *Ralstonia* spp. [27, 28]. OXA-22 is active against benzylpenicillin, cloxacillin and some cephalosporins, while OXA-60 is active against imipenem. Also, *bla*_OXA-569_, *bla*_OXA-570_, *bla*_XA-573_ and *bla*_OXA-574_ are very similar to *bla*_OXA-22_ and *bla*_OXA-60_. Although both our strains harbored a *bla*_OXA-60_ variant (*bla*_OXA-570_ and *bla*_OXA-573_), only isolate WCHRI065162 carrying *bla*_OXA-570_ was resistant to carbapenems. The cloning experiment showed that variant OXA-573 revealed in this study had no carbapenem hydrolase activity. Polymyxin, a cationic polypeptide interacting with the negatively charged phosphate groups in the lipopolysaccharide of the bacterial outer membrane, is the last resort antibiotic against extensively drug-resistant gram-negative bacillus infection. Many pathogens, including *Burkholderia* spp., are inherently resistant to antimicrobial peptides [29]. Members of the *R. pickettii* lineage, including *R. insidiosa*, are intrinsically resistant to colistin [30], which clinicians should be aware of prior to issuing antibiotic prescriptions.

The main limitation of this study is that the source of bloodstream infection in our patients was unknown. Since *Ralstonia* exists widely in the external aqueous environment, a contaminated water supply or parenteral fluid could be the primary source of infection. However, no contamination in our hospital water supply was found during follow up surveillance. Due to our previous limited understanding of *Ralstonia*, no sample from environment at that time was reserved for further investigation. This report serves as an alert for medical workers and researchers to pay more attention to infection caused by *R. insidiosa*.

In conclusion*,* we described two cases of nosocomial bloodstream infection and the microbiological profile of their causable *R. insidiosa* strains. MALDI-TOF MS was cost-effective for rapid species identification. Clinicians should be aware that species *R. insidiosa* is capable of being resistant to many routinely used antibiotics, even carbapenems.

## Abbreviations

ANI: Average nucleotide identity
CLSI: Clinical and Laboratory Standards Institute
MALDI-TOF MS: Matrix assisted laser desorption ionization-time of flight mass spectrometry
MIC: Minimum inhibitory concentrations
SNP: Single nucleotide polymorphism
SS: Secretion system
